# Symptomatic Bradycardia Caused By Premature Atrial Contractions Originating From Right Atrial Appendage

**DOI:** 10.1016/s0972-6292(16)30628-3

**Published:** 2013-06-25

**Authors:** AT Alper, B Gungor, C Turkkan, AI Tekkesin

**Affiliations:** Department of Cardiology, Siyami Ersek Thoracic and Cardiovascular Surgery Center, Training and Research Hospital, Istanbul, Turkey

**Keywords:** bradycardia, premature atrial contraction, radiofrequency ablation

## Abstract

Premature atrial contraction is a common form of supraventricular arrhythmias. In rare cases, severe symptoms other than palpitation may occur. In this report, we present a patient with symptomatic bradycardia which developed secondary to blocked premature atrial contractions and was successfully treated with radiofrequency ablation.

## Introduction

Palpitation is the main complaint in patients with supraventricular arrhythmias. Hemodynamic compromise secondary to high ventricular rates may also occur. In this report, we present a patient with symptomatic bradycardia which developed secondary to blocked premature atrial contractions (PAC) originating from right atrial appendage and was successfully treated with radiofrequency ablation (RFA).

## Case Report

**Figure 1:**
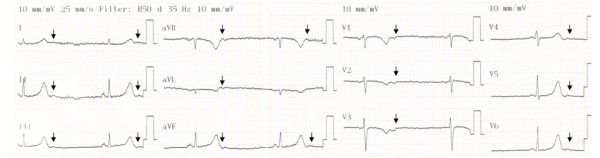
ECG showing bradycardia caused by blocked atrial bigeminy. Arrows indicate non-conducted P waves.

A 35-year-old woman presented to our clinic with dyspnea and fatigue since last two months. Her blood pressure was 90/50 mmHg and her pulse was regular at a rate of 40 beats/min. The physical examination was otherwise normal. Surface ECG was compatible with sinus bradycardia with R-R and P-P wave intervals of 1400 ms and PR interval of 120 ms. But careful observation of the terminal portion of the T waves revealed non-conducted P waves with subsequent prolonged pauses (Figure 1). The morphology of the non-conducted P waves was negative in lead V1,V2, and positive in the inferior leads. A 24-hour Holter monitoring showed more than twenty thousand PACs most of which were not conducted to the ventricle. Her echocardiographic examination and laboratory tests including thyroid function were in normal range.

**Figure 2:**
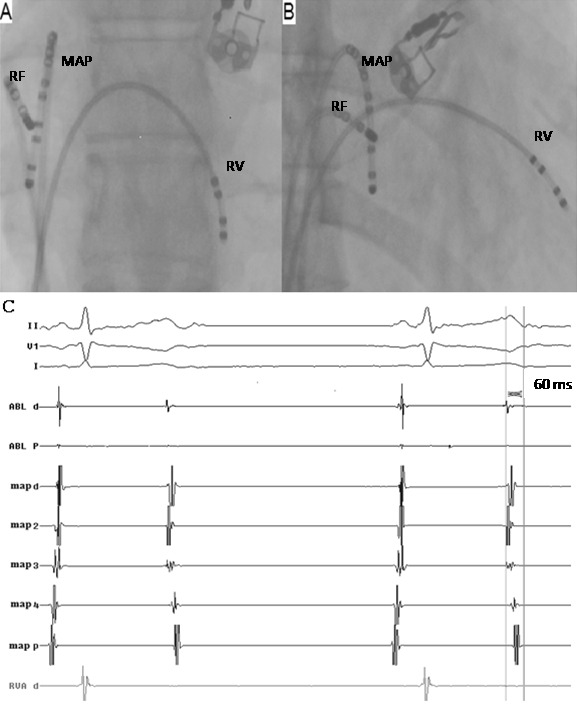
(A) posteroanterior and (B) right anterior oblique flouroscopic views of the catheters during the ablation procedure (C) intracardiac recordings at the site of successful radiofrequency ablation showing that earliest atrial activation precedes the onset of non-conducted P wave by 60 ms in the distal bipolar recordings. Arrows indicate non-conducted P waves. RF, radiofrequency ablation catheter; MAP, mapping catheter; RV, right ventricle catheter.

After obtaining informed consent, the patient was transferred to the electrophysiology laboratory. The procedure was performed with the patient in the fasting, non-sedated state. One 6-French Josephson catheter, one 7-French ablation catheter and one 7-French mapping catheter were advanced through femoral veins and placed at right ventricular apex and right atrium (Figure 2).

**Figure 3:**
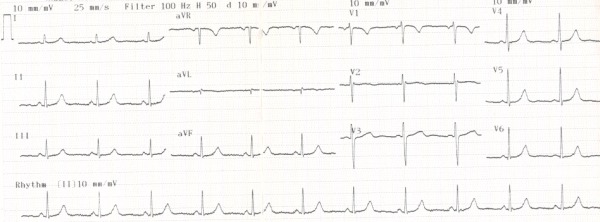
ECG after the ablation procedure showing normal sinus rhythm without any premature atrial contractions.

There were frequent PACs that any maneuver to induce sustained arrhythmia was not needed. Endocardial mapping demonstrated atrioventricular block at suprahisian level and the earliest endocardial activation site was in the right atrial appendage (RAA) where the local intracardiac electrocardiogram was 60 ms prior to the onset of the P waves (Figure 2). Application of the radiofrequency current at this site resulted in termination of the PACs and within 3 seconds. Surface ECG returned to normal sinus rhythm (Figure 3). The rest of the hospitalization was uneventful and the patient did not have any cardiac symptoms during follow-up of 6 months.

## Discussion

Premature atrial contractions are one of the most common forms of arrhythmias. Atrial bigeminy is rarely associated with severe symptoms and palpitation is the only complaint in most of the cases. In our case, the main complaint of the patients was exercise intolerance and fatigue due to bradycardia caused by blocked PACs.

In some cases, non-conducted P waves may superimpose on the T waves of the preceding sinus beats and may be difficult to differentiate from the T and U waves. Thus, the bradycardia may be misdiagnosed as sinoatrial block. Furthermore, blocked atrial bigeminy may be misdiagnosed as second-degree AV block [1]. In these cases unnecessary pacemaker implantation can be avoided by careful ECG examination. In second-degree AV block P-P interval remains constant, and the P wave morphology is unchanged.

Previous case reports have shown that antiarrhythmic drugs [2] and catheter ablation [3] could be alternatives for treatment of atrial bigeminy in symptomatic patients [1-3]. Because of the severe symptoms and high number of PACs, we considered RF ablation as a first line therapy.

In our case the origin of the atrial bigeminy was RAA. In literature, there are no studies about the origins of the atrial bigeminy. But, studies on atrial tachycardia (AT) reported RAA as the site of focal AT in 3.8% of the cases [4]. This study has reported that P wave morphology of AT's originating from RAA is characteristic which is negative in leads V1,V2 and a transition to positivity occurs in the other precordial leads [4]. The P waves in our patient also had similar morphologic features.

## Conclusion

Atrial bigeminy with blocked PACs can result in significant bradycardia with symptoms. Radiofrequency catheter ablation may be considered as the first line of treatment in these patients.

## References

[R1] Gaudio  C (2004). A case of non-conducted atrial bigeminy simulating a second- degree atrioventricular block. A holter ECG diagnosis. Eur Rev Med Pharmacol Sci.

[R2] Veress G (1993). Infra-His blocked premature atrial contractions simulating 2:1 sinoatrial block in a patient with an atrio-His bypass tract. Chest.

[R3] Vora A (2009). ECG of the month. Radiofrequency ablation for bradycardia!. Indian Heart J.

[R4] Freixa X (2008). Characterization of focal right atrial appendage tachycardia. Europace.

